# Bovine-associated non-*aureus* staphylococci suppress *Staphylococcus aureus* biofilm dispersal in vitro yet not through *agr* regulation

**DOI:** 10.1186/s13567-021-00985-z

**Published:** 2021-09-03

**Authors:** Bruno Toledo-Silva, Fernando N. de Souza, Kristien Mertens, Sofie Piepers, Freddy Haesebrouck, Sarne De Vliegher

**Affiliations:** 1 M-Team & Mastitis and Milk Quality Research Unit, Department of Reproduction, Obstetrics and Herd Health, Faculty of Veterinary Medicine, Ghent University, Salisburylaan 133, Merelbeke, 9820 Ghent, Belgium; 2 Veterinary Clinical Immunology Research Group, Department of Clinical Science, Faculty of Veterinary Medicine and Animal Sciences, University of São Paulo, Prof. Orlando Marques de Paiva Av. 87, São Paulo, 05508-270 Brazil; 3 Postgraduate Program in Animal Science, Department of Veterinary Medicine, Federal University of Paraiba, Rodovia PB-079 12, Areia, João Pessoa, 58397-000 Brazil; 4 Department of Pathology, Bacteriology and Avian Diseases, Faculty of Veterinary Medicine, Ghent University, Salisburylaan 133, Merelbeke, 9820 Ghent, Belgium

**Keywords:** Non-*aureus* staphylococci, *Staphylococcus aureus*, biofilm, *agr*, quorum sensing, bovine mastitis

## Abstract

Biofilm formation is a significant virulence factor in *Staphylococcus* (*S.*) *aureus* strains causing subclinical mastitis in dairy cows. A role of environmental signals and communication systems in biofilm development, such as the *agr* system in *S. aureus*, is suggested. In the context of multispecies biofilm communities, the presence of non-*aureus* staphylococci (NAS) might influence *S. aureus* colonization of the bovine mammary gland, yet, such interspecies interactions have been poorly studied. We determined whether 34 *S. chromogenes*, 11 *S. epidermidis*, and 14 *S. simulans* isolates originating from bovine milk samples and teat apices (TA) were able to affect biofilm formation and dispersion of *S. aureus*, and if so, how isolate traits such as the capacity to regulate the *S. aureus agr* quorum sensing system are determinants in this process. The capacity of an *agr*-positive *S. aureus* strain to form biofilm was increased more in the presence of *S. chromogenes* than in the presence of *S. simulans* and *S. epidermidis* isolates and in the presence of NAS isolates that do not harbor biofilm related genes. On the other hand, biofilm dispersion of this particular *S. aureus* strain was suppressed by NAS as a group, an effect that was more pronounced by isolates from TA. Furthermore, the observed effects on biofilm formation and dispersion of the *agr*-positive *S. aureus* strain as well as of an *agr*-negative *S. aureus* strain did not depend on the capacity of NAS to repress the *agr* system.

**Supplementary Information:**

The online version contains supplementary material available at 10.1186/s13567-021-00985-z.

## Introduction

Bovine mastitis, a multifactorial disease-complex resulting from interactions among the host, environment, and typical bacteria, remains the most prevalent and challenging disease on dairy farms worldwide [1]. The non-*aureus* staphylococci (NAS), a heterogeneous group of staphylococcal species other than *Staphylococcus aureus* [2–4] have become the most frequently isolated bacteria from bovine milk samples as revealed by numerous studies [5–9]. They are commonly considered to be minor mastitis pathogens causing only a slight increase of the milk somatic cell count (SCC) [10–12] with no impact on milk yield. Other studies have suggested a protective effect of bovine NAS against, among other bacteria, *S. aureus* [13–17], including the repression of *agr* related virulence factors of *S. aureus* [18, 19].

*Staphylococcus aureus* is considered a contagious mastitis pathogen that enters the mammary gland through the teat canal [20–22]. In most cases, *S. aureus* transmission is associated with chronically infected mammary glands, colonization of skin or mucosal epithelia, nares and (wounded) hocks [22]. Intramammary infections (IMIs) caused by *S. aureus* usually result in subclinical mastitis with poor treatment results based on the current therapies [20, 22]. Biofilm formation is a significant virulence factor of *S. aureus* in case of subclinical mastitis [22, 23].

Biofilms are defined as a structured surface-associated community of bacterial cells (sessile) encompassed by an extracellular matrix [24]. Biofilm development is related to environmental signals and communication systems [25]. In staphylococci it relates to specific gene expression such as the intracellular adhesion locus (*ica*ABCD) [26], the biofilm-associated protein *bap* [27], and the accumulation-associated protein *aap* [28]. In *S. aureus*, the switch from planktonic (free-floating forms) to sessile forms is controlled by quorum-sensing proteins encoded by the *agr*ABCD operon, whose abundance is related to virulence and pathogenicity [29]. Therefore, high levels of *agr* activity is typically associated with dispersal of *S. aureus* biofilms, whereas the *agr* system of cells in a biofilm is predominantly repressed [30, 31].

The thickness and the composition of biofilm might determine its functionality [32] and the persistence of bacteria in the bovine mammary gland [33]. Thicker biofilms are notoriously more difficult to eradicate as they are generally resistant to antibiotic therapy [34] and clearance by host defenses in the mammary gland [32]. Furthermore, multispecies biofilms might play a role in the context of host colonization, since the presence of specific bacteria can either promote or decrease growth of other bacteria [35, 36].

Although a number of studies in literature reported on the protective effect of NAS in the bovine mammary gland, little is known in the context of biofilm formation and whether *agr*-mediated interactions between bovine NAS and *S. aureus* are important. Also, Goetz et al. [32] reported on the capacity of NAS isolates, mainly *S. chromogenes* and *S. simulans*, to reduce biofilm formation and to stimulate dispersion of pre-established biofilm of *S. aureus*. However, the potential mechanisms behind the biofilm-inhibition and dispersion effects of NAS have not yet been identified. Such dual-species interactions might benefit both species with respect to host colonization [37]. Moreover, a robust multispecies biofilm might contribute to the persistence of *S. aureus* infections [38], also in the bovine udder.

Based on the rationale that regulation of the *agr* system can determine biofilm production by *S. aureus*, we hypothesized that *agr*-mediated interactions between NAS and *S. aureus* affect *S. aureus* biofilm formation and dispersal in the bovine mammary gland. In order to investigate the hypothesis, we examined whether bovine-associated NAS originating from milk samples and teat apices (TA) influence biofilm formation and dispersion of *S. aureus* in vitro, and if so, to determine what NAS traits are involved and whether such effects are associated with the regulation of the *S. aureus agr* quorum sensing system.

## Materials and methods

### General study design

First, the presence of genes related to biofilm formation (biofilm genotype) and the capacity to produce biofilm (biofilm phenotype) were mapped for 59 bovine NAS isolates originating from milk or TA (Additional file 1). The capacity to inhibit the growth of *S. aureus* 8325-4 (an *agr*^+^ strain) in vitro (in vitro growth inhibition capacity) and to modulate the *agr* system of *S. aureus* 8325-4 (*agr* interaction capacity) for the 59 NAS isolates was known from previous work [19] (Additional file 1).

Next, it was studied whether the 59 NAS isolates were able to affect *S. aureus* 8325-4 biofilm formation as well as whether they were able to disperse pre-established *S. aureus* 8325-4 biofilm and to what extent this was related to specific NAS traits [species, origin (milk versus TA), biofilm genotype, biofilm phenotype, and in vitro growth inhibition capacity, respectively].

Last, it was studied whether the capacity of the 59 NAS isolates to repress the *agr* system of *S. aureus* was related to biofilm formation and biofilm dispersion by *S. aureus* strains.

### Staphylococcal isolates and traits

The isolates used in this study are listed in Additional file 1*.* Unless otherwise stated, bacteria were grown in Trypticase Soy Broth (TSB) overnight at 37 °C.

#### Species and origin

The 59 NAS isolates were obtained from our repository and represent the three most prevalent species in milk samples and on teat apices of dairy cows and heifers [9, 39]. Forty-five isolates from milk [*S. chromogenes* (*n* = 28), *S. epidermidis* (*n* = 7), and *S. simulans* (*n* = 10)] and 14 isolates from TA were included [*S. chromogenes* (*n* = 6), *S. epidermidis* (*n* = 4), and *S. simulans* (*n* = 4)].

Species identification of all NAS isolates was carried out by matrix-assisted laser desorption/ionization-time of flight (MALDI-ToF) mass spectrometry analysis in which protein fingerprints of the isolates were compared with the commercial databank of bovine reference spectra (Bruker Daltonics), microbial spectra provided by Cameron et al. [40], and additional microbial spectra of field isolates from our lab covering four additional species (*S. jettensis*, *S. lentus*, *S. rostri*, and *S. saprophyticus*).

*Staphylococcus aureus* used in this study include strain 8325-4 (*agr*^+^) [41] and strain 8325-4 Δ*agr* (*agr*^−^) [42].

#### Biofilm genotype

The bacterial DNA of the NAS was extracted [43], with some modifications to identify biofilm associated genes among NAS. In the final step, 3 µL of RNase (1 mg/mL, Roche, Mannheim, Germany) was added. Polymerase chain reaction (PCR) assays were applied to detect the presence of the genes related to biofilm formation (*aap*, *ica*, and *bap*) and quorum sensing (*agr*) according to methodology previously described [27, 28, 44, 45] (Table 1).

**Table 1:** **Oligonucleotide primers used for the detection of genes related to biofilm formation(*bap,*** ***ica,*** **and** ***aap*) and quorum sensing (*agr*)**

Gene	Nucleotide sequence	Amplification conditions	References
*bap*	5′-ACTTAYTRCCHTATATCGAARTAG-3′	94 °C 30 s,	[27]
	5′-GCTGTTGAAGTTAATACTGTACCTGC-3′	57 °C 30 s,	
		72 °C 30 s,	
		30 cycles	
*ica*	5′-CTGTTTCATGGAAACTCC-3′	94 °C 30 s,	[44]
	5′-TCGATGCGATTTGTTCAAACAT-3′	57 °C 30 s,	
		72 °C 30 s,	
		30 cycles	
*aap*	5′-GAAGCACCGAATGTTCCAACTATC-3′	94 °C 30 s,	[28]
	5′-AGTTGGCGGTATATCTATTGTA-3′	54 °C 30 s,	
		72 °C 30 s,	
		30 cycles	
*agr*	5′-CATAGCACTGAGTCCAAGGA-3′	94 °C 30 s,	[45]
	5′-CAATCGGTGACTTAGTAAAATG-3′	55 °C 30 s,	
		72 °C 60 s,	
		30 cycles	

PCR products were analyzed by electrophoresis through 1.5% (wt/vol), previously stained with Ethidium Bromide (Sigma-Aldrich). *Staphylococcus xylosus* ATCC 29971 was used as a positive control for the bap gene and *S. epidermidis* ATCC 35984 for the *aap* and *ica* genes. Positive control for the *agr* system included *S. aureus* strain 8325-4. Isolates with an equal amplicon size to the positive control were considered PCR positive for the particular gene under scrutiny.

The NAS isolates that presented at least one of the four genes were recoded as positive for biofilm-related genes (BG), whereas the absence of all four genes was recoded as negative for biofilm-related genes (NBG; Additional file 1) for statistical analyses.

#### Biofilm phenotype

The potential of the NAS isolates to form biofilm was evaluated according to the assay described by Tremblay et al. [23], with some modifications. Briefly, overnight cultures of *S. aureus* strain 8325-4 [41] and strain 8325-4 Δ*agr* [42] (Additional file 1), and the 59 NAS isolates were adjusted to OD_600_ = 0.2 in TSB and then further diluted 1:100 in TSB supplemented with 0.2% glucose (TSBG). Therefore, an aliquot of 200 μL of each isolate was seeded per well in sterile 96-well polystyrene tissue culture plates at 37 °C for 24 h. After incubation, planktonic cells (supernatant) were removed and the wells were rinsed three times with distilled water. Adherent biofilms were fixed with 95% ethanol for 15 min, and stained with 0.1% (wt/vol) safranin for 20 min. After rinsing three times with distilled water, plates were allowed to dry at room temperature. The stained biofilms were dissolved in 250 μL of 33% (v/v) acetic acid, and optical densities (OD) were measured at 490 nm using Multiskan GO plate reader (Thermo Fisher Scientific, USA). The ability of the isolates to form a biofilm was classified as negative (absorbance at 490 nm, A_490_ < 0.110), weak (A_490_ 0.110–0.500), moderate (A_490_ 0.500–1.500), or strong producers (A_490_ > 1.500) (Additional file 1). Uninoculated wells containing TSB with glucose served as blanks. *Staphylococcus epidermidis* strain ATCC 35984 was used as a strong biofilm forming control strain [46], and *S. epidermidis* strain ATCC 12228 was used as a weak biofilm forming control strain [47].

Each NAS isolate was tested for biofilm production in triplicate on two independent days and results were averaged over the replicates.

All NAS isolates that presented any capacity to produce biofilm (weak, moderate or strong producers) were recoded as positive for biofilm production (BP) for statistical analyses. On the other hand, the absence of biofilm production by NAS was recoded as negative for biofilm production (NBP; Additional file 1).

#### In vitro growth inhibition capacity

The potential growth inhibition of *S. aureus* 8325-4 by the NAS isolates was evaluated before [19] (Additional file 1). Following the methodology described by De Vliegher et al. [13], the pattern of the in vitro growth inhibition of the *S. aureus* strain 8325-4 by the NAS isolates was classified as NAS exhibiting no growth inhibition, partial inhibition, or total inhibition.

The NAS isolates that presented partial or total inhibition were recoded as positive for in vitro growth inhibition of *S. aureus* (GI), whereas no growth inhibition was recoded as negative for in vitro growth inhibition of *S. aureus* (NGI; Additional file 1) for statistical analyses.

#### *agr* repression

Some NAS isolates were able to repress the *agr* system of *S. aureus* 8325-4 by downregulating the *rnaIII* expression [19] (Additional file 1). As described by Canovas et al. [42], the effect of NAS isolates on *S. aureus agr* was classified as none, weak, moderate, or strong. All NAS isolates that showed any capacity to repress *S. aureus agr* (weak, moderate, or strong) were recoded as positive for *agr* repression (RP) for statistical analyses. Conversely, the absence of *agr* repression was recoded as negative for *agr* repression (NRP; Additional file 1).

### *Staphylococcus aureus* biofilm formation

Biofilm interactions of the NAS isolates and *S. aureus* 8325-4 were tested as recently described by Peng et al. [37] with minor adjustments. Overnight cultures of all 59 NAS and *S. aureus* 8325-4 were adjusted to OD_600_ = 0.2 in TSB and then further diluted 1:100 in TSBG. A total of 100 µL of the bacterial suspension(s) were added to wells where either *S. aureus* strains 8325-4 alone (control)*,* or a ratio of 1:1 of *S. aureus* + NAS was added. The plates were incubated for 24 h at 37 °C. The planktonic cells (supernatant) were removed and the biofilms were washed once with sterile water. A total of 200 µL of sterile water was added to the wells and the biofilm cells (*biofilm fractions*) were recovered by scraping the surface with sterile pipette tips, vigorously shaken and serially diluted. The dilutions were plated on a semi-selective agar, mannitol salt agar (MSA, Chapman medium, Oxoid, Aalst, Belgium) for CFU enumeration of *S. aureus* 8325-4 (the outcome variable of interest, CFU/mL). Single-species biofilm of *S. aureus* 8325-4 served as positive control and uninoculated wells containing TSBG served as negative control.

All plates were stained with 0.1% (wt/vol) safranin and the optical density measured (as abovementioned) to confirm the complete detachment of the biofilm fraction from the bottom (data not shown). Each biofilm fraction recovered from the wells was tested for biofilm formation in triplicate on two independent days and results were averaged over the replicates. A CFU/mL lower than numbers recovered from the positive control indicates less *S. aureus* biofilm formation, whereas the opposite is true for higher values of CFU/mL.

### *Staphylococcus aureus* biofilm dispersion

To evaluate the potential of the NAS isolates to disperse the preformed biofilm of *S. aureus,* the biofilm dispersion assay was performed [32, adapted]. Briefly, *S. aureus* strain 8325-4 biofilms were grown as described in the Section “Biofilm phenotype”, and after the 24-h incubation, planktonic cells were discarded. The pre-formed *S. aureus* biofilms were washed once with sterile water. After, overnight cultures of all 59 NAS were adjusted in TSBG as abovementioned (Section “Biofilm phenotype”) and 200 µL of the suspensions were added directly to the *S. aureus* pre-formed biofilms. Plates were incubated for another 24 h at 37 °C. Finally, the planktonic cells were removed (*dispersed fractions*) and serially diluted. The dilutions were plated on MSA plates for CFU enumeration of *S. aureus* 8325-4 (the outcome variable of interest). Single-species biofilm of *S. aureus* 8325-4 served as positive control and uninoculated wells containing TSBG served as negative control.

All plates were stained with 0.1% (wt/vol) safranin and the optical density measured (as abovementioned) to confirm the complete detachment of the biofilm fraction from the bottom (data not shown). Each dispersion fraction recovered from the wells was tested for biofilm dispersion in triplicate on two independent days and results were averaged over the replicates. A CFU/mL lower than numbers recovered from the positive control indicates less *S. aureus* biofilm dispersion, whereas the opposite is true for higher values of CFU/mL.

### Statistical analyses

All statistical analyses were performed using SPSS v.27.0 (IBM Corp., Armonk, NY, USA) and *P* ≤ 0.05 was considered significant. One-way analysis of variance (ANOVA) was used to determine whether the biofilm formation as well as biofilm dispersion of *S. aureus* 8325-4 (CFU/mL; normally distributed as assessed through Q-Q plots and Kolmogorov-Smirnova test of normality) differed: (1) between NAS according to their species (3 levels: *S. chromogenes, S. epidermidis*, and *S. simulans*), (2) between NAS isolates originating from the two different habitats (2 levels: milk or TA), and (3) between NAS harboring biofilm-related genes or not [2 levels: positive for at least one gene (BG) and negative (NBG); biofilm genotype), between NAS with capacity to produce biofilms themselves or not [2 levels: positive for any capacity to produce biofilm (BP) and negative (NBP); biofilm phenotype], between NAS with capacity to inhibit growth of *S. aureus* in vitro or not [2 levels: positive for partial or total growth inhibition of *S. aureus* (GI) and negative (NGI)]. In addition, one-way ANOVA was carried out to verify whether the biofilm formation and dispersion of *S. aureus* 8325-4 and 8325-4 Δ*agr* (CFU/mL) was affected by the NAS capacity to suppress the *agr* system of *S. aureus* 8325-4 [2 levels: positive for any suppression of *S. aureus agr* (RP) and negative (NRP)].

Least significant difference (LSD) test was used to compare with the controls and post-hoc Bonferroni corrections were applied for all other comparisons.

## Results

### Non-*aureus* staphylococcal traits

Biofilm-related genes were identified in 44.1% (15/34) of the *S. chromogenes* isolates, whereas this was in 100% (11/11) of the *S. epidermidis* isolates and in 85.7% (12/14) of the *S. simulans* isolates (Additional file 1). The capacity to produce biofilm was observed in 8.8% (3/34), 72.7% (8/11) and 35.7% (5/14) of the *S. chromogenes*, *S. epidermidis* and *S. simulans* isolates, respectively (Additional file 1). Also, 100% (34/34) of the *S. chromogenes* isolates has the capacity to inhibit growth of *S. aureus* in vitro at least partially, whilst this was true for 45.4% (5/11) and 100% (14/14) of the *S. epidermidis* and *S. simulans* isolates, respectively [19] (Additional file 1). Seventy-nine (27/34), 27.3 (3/11) and 100% (14/14) of the *S. chromogenes*, *S. epidermidis*, and *S. simulans* isolates, respectively, repressed the *S. aureus agr* system [19] (Additional file 1).

### *Staphylococcus aureus* biofilm formation

**Figure 1:**
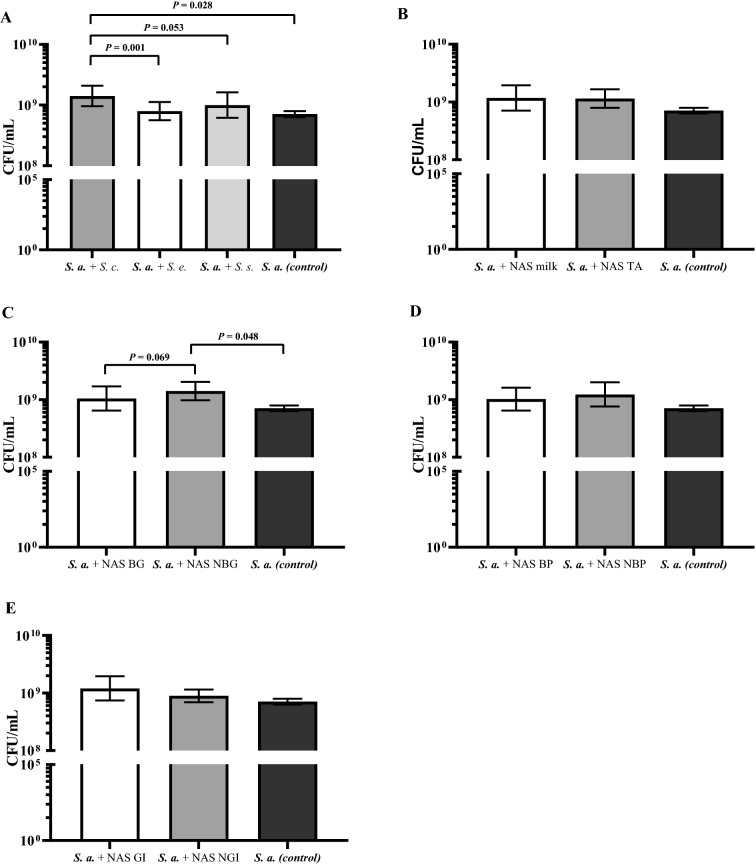
**Effect of the 59 non-*aureus Staphylococcus* (NAS) isolates on *S. aureus* (*S. a*.) biofilm formation (CFU/mL of the biofilm fraction).** For dual-species biofilms, *S. aureus* 8325-4 (*agr* +) was co-cultured 24 h together with **A** *S. chromogenes* (*S. c.*), *S. epidermidis* (*S. e.*) and *S. simulans* (*S. s.*); **B** originating from milk or teat apices (TA); **C** harboring biofilm-related genes (BG) or not (NBG); **D** with the capacity to produce biofilm themselves (BP) or not (NBP); **E** with the capacity to inhibit the growth of *S. aureus* (GI) or not (NGI) and compared to biofilm formed by *S. aureus* alone (black bars).

Biofilm formation of *S. aureus* 8325-4 was quantified in the presence and absence (control) of NAS (Figure 1).

Overall, NAS as a group (comprising *S. chromogenes, S. epidermidis,* and *S. simulans*) did not influence *S. aureus* 8325-4 biofilm formation. Also, neither the origin of the NAS isolates (milk versus TA; Figure 1B) nor the biofilm phenotype (Figure 1D), nor the in vitro growth inhibition capacity of NAS (Figure 1E) were related to *S. aureus* biofilm formation. Still, *S. chromogenes* stimulated *S. aureus* biofilm formation (*P* = 0.028; Figure 1A). Also, the stimulation of the *S. aureus* biofilm formation was more pronounced for *S. chromogenes* than for *S. epidermidis* (*P* = 0.001) and *S. simulans* (*P* = 0.053; Figure 1A). Also, NAS not carrying biofilm genes stimulated *S. aureus* biofilm formation (*P* = 0.048), which was not true for NAS carrying biofilm genes although there was a strong tendency (*P* = 0.069; Figure 1C).

**Figure 2:**
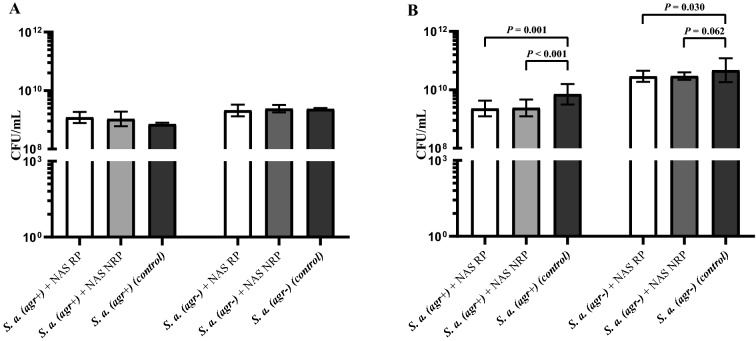
**Effect of the 59 non-*aureus Staphylococcus* (NAS) on biofilm formation (A) and dispersion (B) of *S. aureus* (CFU/mL of the biofilm and dispersed fraction, respectively) taking into account the capacity of NAS to regulate the *agr* system of *S. aureus*** [19]**.** For both assays, *S*. *aureus* 8325-4 [*S. a.* (*agr* +)] and 8325-4 Δ*agr* [*S. a.* (*agr*-)] biofilms (**A**) or pre-established biofilms (**B**) were co-cultured together with NAS isolates that are able to repress the *agr* system of *S. aureus* (RP) or not (NRP) and compared to biofilm formed by *S. aureus* alone (black bars).

The capacity of *S. aureus* to form biofilm did not depend on the capacity of NAS to repress the *S. aureus agr* system, as evidenced for both an *agr*-positive and *agr*-negative *S. aureus* strain (Figure 2A).

### *Staphylococcus aureus* biofilm dispersion

**Figure 3:**
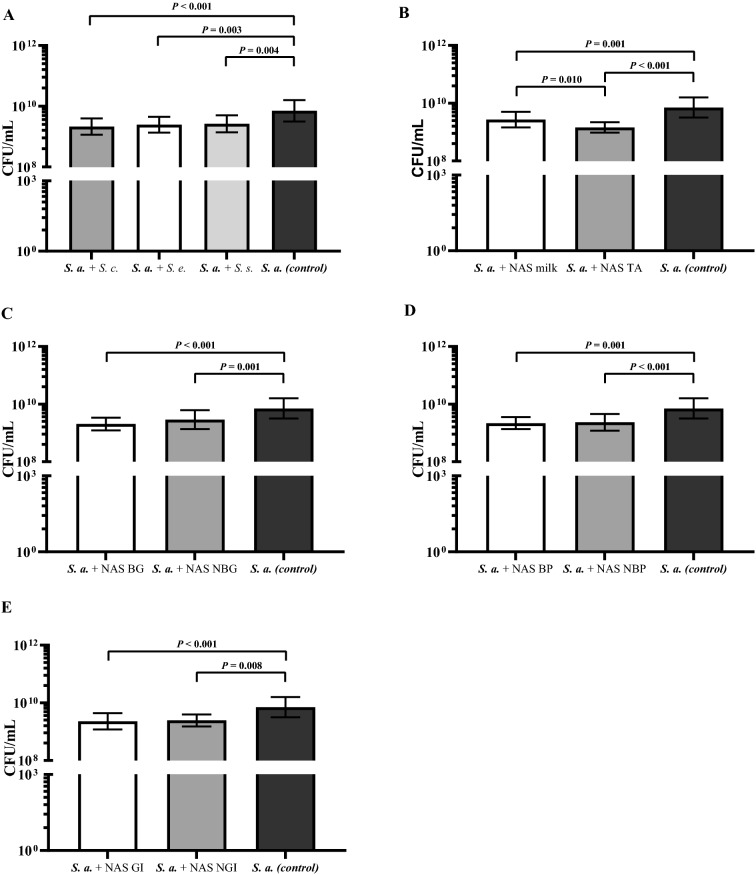
**Effect of the 59 non-*aureus* staphylococcus (NAS) isolates on *S. aureus* (*S. a.*) biofilm dispersion (CFU/mL of the dispersed fraction).** For dispersal of biofilms, pre-established (24 h) *S. a.* 8325-4 (*agr* +) was co-cultured 24 h together with **A** *S. chromogenes* (*S. c.*), *S. epidermidis* (*S. e.*) and *S. simulans* (*S. s.*); **B** originating from milk or teat apices (TA); **C** harboring biofilm-related genes (BG) or not (NBG); **D** with the capacity to produce biofilm themselves (BP) or not (NBP); **E** with the capacity to inhibit the growth of *S. aureus* (GI) or not (NGI) and compared to biofilm formed by *S. aureus* alone (black bars).

The quantitative determination (CFU/mL) of biofilm dispersion of *S. aureus* 8325-4 in the presence and absence (control) of NAS isolates is shown in Figure 3.

Overall, NAS as a group (comprising *S. chromogenes*, *S. epidermidis*, and *S. simulans*) significantly suppressed *S. aureus* 8325-4 biofilm dispersion, although this was more pronounced in NAS isolates originating from TA than from milk (*P* = 0.01; Figure 1B). Neither the NAS species (Figure 1A), nor the biofilm genotype (Figure 1C), nor the biofilm phenotype (Figure 1D), nor in vitro growth inhibition capacity of NAS (Figure 1E) were associated with *S. aureus* biofilm dispersion.

The ability of NAS to suppress *S. aureus* biofilm dispersion did not depend on their capacity to repress the *agr* system, as evidenced by the fact that the suppression was observed for both an *agr*-positive and *agr*-negative *S. aureus* strain (Figure 2B).

## Discussion

We showed that *S. chromogenes* stimulates *S. aureus* biofilm formation. The latter effect is most likely, at least partially, explained by the fact that *S. chromogenes* isolates were seldomly carrying biofilm genes or producing biofilms themselves, an association that was also evident from our experiments, a finding that is discussed later. On the other hand, all NAS isolates, especially those originating from TA, were able to suppress *S. aureus* biofilm dispersion*.* Interestingly, neither the biofilm formation nor the biofilm dispersion of *S. aureus* in the presence of bovine NAS isolates was regulated via the *S. aureus agr* quorum sensing system.

As was expected, the capacity of the NAS isolates to form biofilm themselves varied among the species, with *S. epidermidis* isolates presenting the highest ability to do so (72.7%). Conversely, a study [23] reported that *S. epidermidis* displayed the lowest ability to produce biofilm among the same three NAS species as used in our study, although the overall percentage of isolates forming biofilm was quite similar to ours (69.3%). The percentage of *S. chromogenes* (8.8%) as well as *S. simulans* isolates (35.7%) able to form biofilm in our study was considerably lower than the percentage observed by the other study [23], with respectively 84.7% and 84.9% of their isolates being biofilm formers. The media used for the biofilm assays (BHI vs. TSB) could be one of the reasons for the differences observed between studies. As well important, in the other study [23] all the NAS isolates originated only from bovine milk, whereas ours from both bovine milk and TA. Still, the 44.1% of *S. chromogenes* isolates harboring at least one of the biofilm-related genes in our study was also much lower than the 82% found by Tremblay et al. [23].

Interestingly, the biofilm phenotype observed in our isolates was not well-correlated with the identification of genes associated with biofilm formation (*bap*, *ica*, *aap*, and *agr*) in the same isolates. We demonstrated that the carriage of genes was detected in the majority of NAS originating from milk (63.2%) yet that only half of these isolates actually produced biofilm. Piessens et al. [48] also observed that only nine out of the 30 *bap*- and/or *icaA*-positive NAS isolates produced biofilm in the phenotypic assay. However, other study [23] reported that a combination of multiple genes, such as *icaA-bap-aap* and *icaA-aap*, was associated with a greater ability to form a biofilm. In our study, oddly, none of the NAS isolates possessed *bap* or *ica* genes, yet, seven out of nine *aap-agr* positive *S. epidermidis* isolates were able to produce biofilm. The failure to detect *bap* or *ica* genes in the PCR analyses may occur, but does not necessarily imply the inability to form a biofilm by NAS [27, 49], as other factors can mediate the biofilm-forming process. Additionally, it was reported [48] that so-called environmental NAS species (only causing IMI sporadically), such as *S. epidermidis*, *S. sciuri*, and *S. xylosus*, are a more significant reservoir of biofilm-related genes than so-called contagious NAS species (primarily causing IMI).

Our findings regarding the effect of NAS on the *S. aureus* biofilm formation and dispersion were in contrast with the observations revealed in a recent study [32]. In the latter study, a reduction in biofilm formation as well as an increase in dispersion of pre-established biofilm of *S. aureus* by bovine NAS, especially by *S. chromogenes* and *S. simulans* isolates, was reported. Unlike our study though, their *S. chromogenes* isolates were essentially weak-biofilm producers, whereas ours rarely did produce biofilm. Strikingly, the *S. chromogenes* isolates in our collection were less likely to carry biofilm-related genes. Also, the capacity of bovine NAS isolates to stimulate *S. aureus* biofilm formation was more extended in NAS isolates that did not carry biofilm-related genes. Although the presence of such genes and the capacity to form biofilm were not well-correlated, we believe this finding explains, at least partially, the *S. chromogenes* effect and that the discordant results might be attributed to differences in intrinsic traits of the NAS isolates as well as to the combination of *S. aureus* and NAS species. Unfortunately, in-depth information regarding the traits of the NAS isolates used was not informed by Goetz et al. [32].

In addition to the effect of *S. chromogenes* isolates on the *S. aureus* biofilm formation, the dispersion of the pre-established biofilm of *S. aureus* by the bovine NAS isolates was suppressed, even more so by TA isolates (including the isolate *S. c*. 29—a strong in vitro growth inhibitor). Some studies have reported advantages provided by living in a biofilm community, which may result in, for example, decreased antibiotic susceptibility and protection against immune defenses [50, 51]. Furthermore, according to our previous findings [19], the same NAS isolates originating from TA, except for one *S. epidermidis* isolate, demonstrated in vitro *S. aureus* growth inhibition*.* However, the capacity of the bovine NAS isolates to inhibit in vitro the growth of *S. aureus* did not play a role in the biofilm regulation of *S. aureus* under the conditions tested in our study*.* Likewise, other study [32] observed no marked growth inhibition when *S. aureus* isolates were grown with the *S. chromogenes* and *S. simulans* isolates that had the strongest biofilm-inhibition activity.

Different interactions between NAS and *S. aureus* colonizing the bovine mammary gland have been suggested [18, 19], including the competitive behavior between NAS and *S. aureus* in multispecies biofilm communities [32]. In a previous study using the same NAS isolates, we demonstrated that NAS have the capacity to repress the *agr* system of *S. aureus* [19]. Furthermore, based on the concept that *agr* might coordinate both biofilm formation and dispersal [31], we verified for the first time whether bovine NAS isolates originating from milk and TA might affect biofilm formation or dispersion of *S. aureus* by regulation of the *agr* system Although it was previously reported that the repression of the *agr* system would be necessary to form a biofilm, whereas the reactivation of the *agr* system in established biofilms would trigger its dispersion [31], our NAS isolates not only suppressed biofilm dispersion of the *S. aureus* strain with a functional *agr* system (8325-4) but also from the *agr* mutant strain (8325-4 Δ*agr*). Also, the effect of NAS on the biofilm dispersion of both the parent and mutant *S. aureus* strain (8235-4 and 8325-4 Δ*agr*) further reinforced the hypothesis that the bovine NAS isolates can coordinate biofilm formation and dispersion of *S. aureus* by other mechanisms rather than regulation of *agr* quorum sensing system.

Some authors have discussed that the presence or absence of bovine NAS isolates sharing the same niche as *S. aureus* may play a role in the host colonization [18], whereas others [37, 52] observed a robust biofilm formation by NAS plus *S. aureus* in isolates from pigs, with no evident out-competition of one over the other species when growing in physical contact. According to Otto [53], in fact more than one mechanism could be involved in the biofilm formation and dispersion of staphylococci species, such as the bacterial social behavior (cooperation and/or competition), the molecular mechanism (e.g., bacterial signaling, coaggregation, or co-metabolism) [51], the immune response of the host (bovine mammary gland immune response), and both intrinsic and acquired traits of the isolates involved in the dual-species bacterial biofilms [54]. Nevertheless, as biofilms are extremely hard to eradicate by both the host and by antimicrobial therapies, these dual-species interactions would allow both species to persist in a colonizing state more robustly in one hand, but on the other hand would also provide a constant reservoir for possible *S. aureus* chronic infections [38]. Still, the reduction of dispersion, as was evident in our results, could potentially prevent *S. aureus* to spread and colonize other niches.

In conclusion, we demonstrated that *S. chromogenes* as well as NAS isolates in which no biofilm-related genes were demonstrated, stimulate biofilm formation of *S. aureus.* As well, bovine NAS isolates were effective in suppressing biofilm dispersion of *S. aureus.* Interestingly, the habitat of the NAS isolates seems to play an important role in the dispersion of the *S. aureus* biofilm, suggesting strain differences related to niche adaptation. Importantly, our findings highlight the existing interactions between bovine-related NAS and *S. aureus* in the biofilm communities and suggest as well that the *S. aureus* biofilm formation and dispersion, though not through regulation of the *agr* quorum sensing system, can be affected by (some) NAS (species). The mechanisms that coordinate and determine the biofilm formation and dispersion of *S. aureus* in the presence of NAS isolates have yet to be identified. Our findings contribute to the knowledge on biofilm formation and dispersion of *S. aureus* and may lead to new preventive or treatment measures, especially for chronic subclinical mastitis caused by this bacterium in dairy cows.

## Supplementary Information

**Additional file 1. Identification and traits of bovine** ***S. chromogenes*** **(S. c.), S** ***. epidermidis*** **(S. e.),** ***S. simulans*** **(S. s.), and** ***Staphylococcus aureus*** **(S. a.).**

## Data Availability

The data on which the conclusions of the manuscript rely are presented in the main paper and additional files.

## Abbreviations

*agr*: accessory gene regulator
*aap*: accumulation-associated protein
*bap*: biofilm-associated protein
BG: positive for biofilm-related genes
BP: positive for biofilm production
CFU: colony forming units
GI: positive for in vitro growth inhibition of *S. aureus*
h: hours
*ica*ABCD: intracellular adhesion locus
IMI: intramammary infection
MALDI-ToF: matrix assisted laser desorption/ionization time-of-fight mass spectrometry
MSA: mannitol salt agar
NAS: non-*aureus* staphylococci
NBG: negative for biofilm-related genes
NBP: negative for biofilm production
NGI: negative for in vitro growth inhibition of *S. aureus*
NRP: negative for *agr* repression
Neg.: negative
OD: optical density
PCR: polymerase chain reaction
Pos.: positive
*S. a.*: *Staphylococcus aureus*
*S. c.*: *Staphylococcus chromogenes*
*S. e.*: *Staphylococcus epidermidis*
*S. s.*: *Staphylococcus simulans*
RP: positive for *agr* repression
TA: teat apex
TSB: tryptic soy broth
SCC: quarter somatic cell count
