# Supplementary material for: Bovine-associated non-aureus staphylococci suppress Staphylococcus aureus biofilm dispersal in vitro yet not through agr regulation
Source: Vet Res. 2021 Sep 3;52:114. doi: 10.1186/s13567-021-00985-z (PMC8414718; doi:10.1186/s13567-021-00985-z)
Supplement: Supplementary file 1 — Additional file 1. Identification and traits of bovine S. chromogenes (S. c.), S . epidermidis (S. e.), S. simulans (S. s.), and Staphylococcus aureus (S. a.). [file 13567_2021_985_MOESM1_ESM.docx]

**Additional file 1. Identification and traits of bovine *S. chromogenes* (*S. c.*), *S. epidermidis* (*S. e.*), *S. simulans* (*S. s.*), and *Staphylococcus aureus* (*S. a.).***

¹ Obtained from Supré et al. [8];

^2^ Obtained from De Vliegher et al. [13];

^3^ Obtained from Novick [41];

^4^ Obtained from Canovas et al. [42];

^5^ Neg. = Negative; Pos. = Positive;

^6^ Recoded as a dichotomous variable for statistical analyses, where BG = positive for biofilm-related genes; NBG = negative for biofilm-related genes; BP = positive for biofilm production; NBP = negative for biofilm production; GI = positive for in vitro growth inhibition of *S. aureus*; NGI = negative for in vitro growth inhibition of *S. aureus*; RP = positive for *agr* repression; NRP = negative for *agr* repression;

^7^ Results from Toledo-Silva et al. [19].

| Species and isolates | Origin | Biofilm genotype^5^ | | | | Biofilm genotype recoded^6^ | Biofilm phenotype | Biofilm phenotype recoded^6^ | *S. aureus* growth inhibition^7^ | *S. aureus* growth inhibition capacity recoded^6^ | *agr* repression^7^ | *agr* repression recoded^6^ |
| --- | --- | --- | --- | --- | --- | --- | --- | --- | --- | --- | --- | --- |
|  |  | *bap* | *ica* | *aap* | *agr* |  |  |  |  |  |  |  |
| NAS |  |  |  |  |  |  |  |  |  |  |  |  |
| *S. chromogenes (S. c.)* | |  |  |  |  |  |  |  |  |  |  |  |
| *S. c. 1 - “IM”^1^* | Milk | Neg. | Neg. | Pos. | Neg. | BG | Neg. | NBP | Partial | GI | Weak | RP |
| *S. c. 2* | Milk | Neg. | Neg. | Neg. | Pos. | BG | Neg. | NBP | Partial | GI | Weak | RP |
| *S. c. 3* | Milk | Neg. | Neg. | Neg. | Neg. | NBG | Neg. | NBP | Partial | GI | Strong | RP |
| *S. c. 4* | Milk | Neg. | Neg. | Neg. | Pos. | BG | Neg. | NBP | Partial | GI | None | NRP |
| *S. c. 5* | Milk | Neg. | Neg. | Neg. | Pos. | BG | Neg. | NBP | Partial | GI | Moderate | RP |
| *S. c. 6* | Milk | Neg. | Neg. | Neg. | Pos. | BG | Neg. | NBP | Partial | GI | Weak | RP |
| *S. c. 7* | Milk | Neg. | Neg. | Neg. | Pos. | BG | Neg. | NBP | Partial | GI | None | NRP |
| *S. c. 8* | Milk | Neg. | Neg. | Neg. | Neg. | NBG | Neg. | NBP | Partial | GI | None | NRP |
| *S. c. 9* | Milk | Neg. | Neg. | Pos. | Neg. | BG | Neg. | NBP | Partial | GI | None | NRP |
| *S. c. 10* | Milk | Neg. | Neg. | Neg. | Neg. | NBG | Neg. | NBP | Partial | GI | Strong | RP |
| *S. c. 11* | Milk | Neg. | Neg. | Neg. | Neg. | NBG | Neg. | NBP | Partial | GI | None | NRP |
| *S. c. 12* | Milk | Neg. | Neg. | Neg. | Neg. | NBG | Neg. | NBP | Partial | GI | Weak | RP |
| *S. c. 13* | Milk | Neg. | Neg. | Pos. | Neg. | BG | Neg. | NBP | Partial | GI | Weak | RP |
| *S. c. 14* | Milk | Neg. | Neg. | Neg. | Neg. | NBG | Neg. | NBP | Partial | GI | Moderate | RP |
| *S. c. 15* | Milk | Neg. | Neg. | Neg. | Neg. | NBG | Neg. | NBP | Partial | GI | Weak | RP |
| *S. c. 16* | Milk | Neg. | Neg. | Neg. | Neg. | NBG | Weak | BP | Partial | GI | Strong | RP |
| *S. c. 17* | Milk | Neg. | Neg. | Neg. | Neg. | NBG | Neg. | NBP | Partial | GI | Weak | RP |
| *S. c. 18* | Milk | Neg. | Neg. | Neg. | Neg. | NBG | Weak | BP | Partial | GI | Moderate | RP |
| *S. c. 19* | Milk | Neg. | Neg. | Neg. | Neg. | NBG | Neg. | NBP | Partial | GI | Strong | RP |
| *S. c. 20* | Milk | Neg. | Neg. | Neg. | Neg. | NBG | Neg. | NBP | Partial | GI | Strong | RP |
| *S. c. 21* | Milk | Neg. | Neg. | Neg. | Neg. | NBG | Neg. | NBP | Partial | GI | Moderate | RP |
| *S. c. 22* | Milk | Neg. | Neg. | Neg. | Neg. | NBG | Neg. | NBP | Partial | GI | Strong | RP |
| *S. c. 23* | Milk | Neg. | Neg. | Neg. | Neg. | NBG | Neg. | NBP | Partial | GI | Weak | RP |
| *S. c. 24* | Milk | Neg. | Neg. | Neg. | Neg. | NBG | Neg. | NBP | Partial | GI | Weak | RP |
| *S. c. 25* | Milk | Neg. | Neg. | Neg. | Neg. | NBG | Moderate | BP | Partial | GI | Strong | RP |
| *S. c. 26* | Milk | Neg. | Neg. | Neg. | Neg. | NBG | Neg. | NBP | Partial | GI | Strong | RP |
| *S. c. 27* | Milk | Neg. | Neg. | Pos. | Neg. | BG | Neg. | NBP | Partial | GI | Weak | RP |
| *S. c. 28* | Milk | Neg. | Neg. | Neg. | Neg. | NBG | Neg. | NBP | Partial | GI | None | NRP |
| *S. c. 29 - “TA”^2^* | Teat apex | Neg. | Neg. | Pos. | Neg. | BG | Neg. | NBP | Total | GI | Strong | RP |
| *S. c. 30* | Teat apex | Neg. | Neg. | Pos. | Neg. | BG | Neg. | NBP | Partial | GI | Weak | RP |
| *S. c. 31* | Teat apex | Neg. | Neg. | Pos. | Neg. | BG | Neg. | NBP | Partial | GI | None | NRP |
| *S. c. 32* | Teat apex | Neg. | Neg. | Pos. | Neg. | BG | Neg. | NBP | Partial | GI | Moderate | RP |
| *S. c. 33* | Teat apex | Neg. | Neg. | Pos. | Neg. | BG | Neg. | NBP | Partial | GI | Strong | RP |
| *S. c. 34* | Teat apex | Neg. | Neg. | Pos. | Neg. | BG | Neg. | NBP | Partial | GI | Strong | RP |
| *S. epidermidis (S. e.)* | |  |  |  |  |  |  |  |  |  |  |  |
| *S. e. 1* | Milk | Neg. | Neg. | Neg. | Pos. | BG | Neg. | NBP | Partial | GI | None | NRP |
| *S. e. 2* | Milk | Neg. | Neg. | Pos. | Pos. | BG | Weak | BP | Partial | GI | None | NRP |
| *S. e. 3* | Milk | Neg. | Neg. | Pos. | Pos. | BG | Neg. | NBP | None | NGI | None | NRP |
| *S. e. 4* | Milk | Neg. | Neg. | Pos. | Pos. | BG | Weak | BP | None | NGI | None | NRP |
| *S. e. 5* | Milk | Neg. | Neg. | Pos. | Pos. | BG | Weak | BP | None | NGI | Weak | RP |
| *S. e. 6* | Milk | Neg. | Neg. | Pos. | Pos. | BG | Weak | BP | None | NGI | Weak | RP |
| *S. e. 7* | Milk | Neg. | Neg. | Pos. | Pos. | BG | Neg. | NBP | None | NGI | None | NRP |
| *S. e. 8* | Teat apex | Neg. | Neg. | Pos. | Pos. | BG | Weak | BP | Partial | GI | None | NRP |
| *S. e. 9* | Teat apex | Neg. | Neg. | Pos. | Pos. | BG | Weak | BP | Partial | GI | None | NRP |
| *S. e. 10* | Teat apex | Neg. | Neg. | Pos. | Neg. | BG | Strong | BP | Partial | GI | None | NRP |
| *S. e. 11* | Teat apex | Neg. | Neg. | Pos. | Pos. | BG | Weak | BP | None | NGI | Weak | RP |
| *S. simulans (S. s.)* | |  |  |  |  |  |  |  |  |  |  |  |
| *S. s. 1* | Milk | Neg. | Neg. | Pos. | Neg. | BG | Neg. | NBP | Partial | GI | Moderate | RP |
| *S. s. 2* | Milk | Neg. | Neg. | Pos. | Neg. | BG | Neg. | NBP | Partial | GI | Strong | RP |
| *S. s. 3* | Milk | Neg. | Neg. | Neg. | Neg. | NBG | Neg. | NBP | Partial | GI | Strong | RP |
| *S. s. 4* | Milk | Neg. | Neg. | Neg. | Neg. | NBG | Neg. | NBP | Partial | GI | Moderate | RP |
| *S. s. 5* | Milk | Neg. | Neg. | Pos. | Neg. | BG | Neg. | NBP | Partial | GI | Moderate | RP |
| *S. s. 6* | Milk | Neg. | Neg. | Pos. | Neg. | BG | Neg. | NBP | Partial | GI | Moderate | RP |
| *S. s. 7* | Milk | Neg. | Neg. | Pos. | Neg. | BG | Neg. | NBP | Partial | GI | Strong | RP |
| *S. s. 8* | Milk | Neg. | Neg. | Pos. | Neg. | BG | Neg. | NBP | Partial | GI | Weak | RP |
| *S. s. 9* | Milk | Neg. | Neg. | Pos. | Neg. | BG | Neg. | NBP | Partial | GI | Moderate | RP |
| *S. s. 10* | Milk | Neg. | Neg. | Pos. | Neg. | BG | Weak | BP | Partial | GI | Strong | RP |
| *S. s. 11* | Teat apex | Neg. | Neg. | Pos. | Neg. | BG | Weak | BP | Total | GI | Moderate | RP |
| *S. s. 12* | Teat apex | Neg. | Neg. | Pos. | Neg. | BG | Weak | BP | Partial | GI | Moderate | RP |
| *S. s. 13* | Teat apex | Neg. | Neg. | Pos. | Neg. | BG | Moderate | BP | Partial | GI | Moderate | RP |
| *S. s. 14* | Teat apex | Neg. | Neg. | Pos. | Neg. | BG | Weak | BP | Partial | GI | Moderate | RP |
| *S. aureus (S. a.)* | |  |  |  |  |  |  |  |  |  |  |  |
| *S. a.* 8325-4^3^ | Wild-type (*agr group I*) |  |  |  |  |  | Strong | BP |  |  |  |  |
| *S. a.* 8325–4 Δ*agr^4^* | Transduction from *S. aureus*  RN6911 (*agr*-) |  |  |  |  |  | Strong | BP |  |  |  |  |
